# Electronic Circular Dichroism Spectra of DNA Quadruple Helices Studied by Molecular Dynamics Simulations and Excitonic Calculations including Charge Transfer States

**DOI:** 10.3390/molecules26164789

**Published:** 2021-08-07

**Authors:** Haritha Asha, James A. Green, Lara Martinez-Fernandez, Luciana Esposito, Roberto Improta

**Affiliations:** 1 CNR—Consiglio Nazionale delle Ricerche, Istituto di Biostrutture e Bioimmagini (IBB-CNR), Via Mezzocannone 16, I-80136 Napoli, Italy; haritha.asha@ibb.cnr.it (H.A.); james.green@ibb.cnr.it (J.A.G.); luciana.esposito@cnr.it (L.E.); 2 Departamento de Química, Facultad de Ciencias and IADCHEM (Institute for Advanced Research in Chemistry), Universidad Autónoma de Madrid, Cantoblanco, 28049 Madrid, Spain; lara.martinez@uam.es

**Keywords:** Guanine, telomere, quantum mechanical calculations, excited electronic states, excitonic model, charge transfer states

## Abstract

We here investigate the Electronic Circular Dichroism (ECD) Spectra of two representative Guanine-rich sequences folded in a Quadruple helix (GQ), by using a recently developed fragment diabatisation based excitonic model (FrDEx). FrDEx can include charge transfer (CT) excited states and consider the effect of the surrounding monomers on the local excitations (LEs). When applied to different structures generated by molecular dynamics simulations on a fragment of the human telomeric sequence (Tel21/22), FrDEx provides spectra fully consistent with the experimental one and in good agreement with that provided by quantum mechanical (QM) method used for its parametrization, i.e., TD-M05-2X. We show that the ECD spectrum is moderately sensitive to the conformation adopted by the bases of the loops and more significantly to the thermal fluctuations of the Guanine tetrads. In particular, we show how changes in the overlap of the tetrads modulate the intensity of the ECD signal. We illustrate how this correlates with changes in the character of the excitonic states at the bottom of the $L_{a}$ and $L_{b}$ bands, with larger LE and CT involvement of bases that are more closely stacked. As an additional test, we utilised FrDEx to compute the ECD spectrum of the monomeric and dimeric forms of a GQ forming sequence T30695 (5${}^{\prime }$TGGGTGGGTGGGTGGG3${}^{\prime }$), i.e., a system containing up to 24 Guanine bases, and demonstrated the satisfactory reproduction of the experimental and QM reference results. This study provides new insights on the effects modulating the ECD spectra of GQs and, more generally, further validates FrDEx as an effective tool to predict and assign the spectra of closely stacked multichromophore systems.

## 1. Introduction

**Figure 1:**
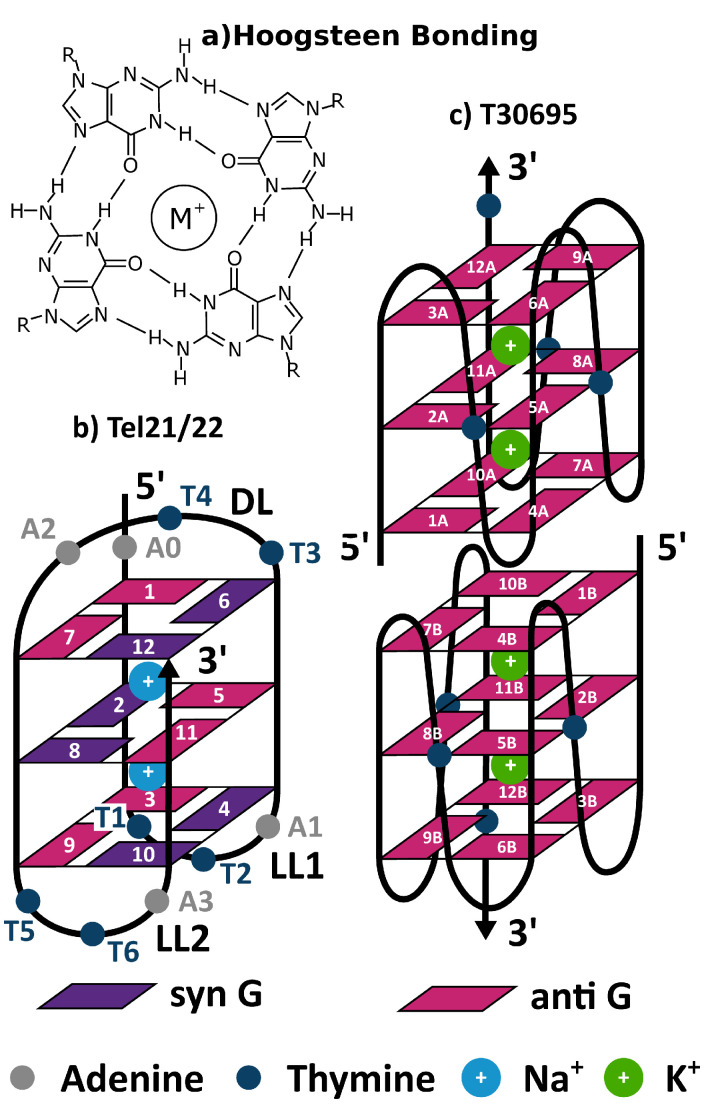
Schematic drawing of (**a**) Hoogsteen bonding arrangement of four guanines within a tetrad and the guanine quadruplex structures of (**b**) Tel21/22 (antiparallel) and (**c**) T30695 (parallel dimer). Only the guanine bases are numbered and labelled for T30695, both for monomers A and B. The adenine and thymine bases are numbered in addition to the guanines for Tel21/22, with Tel21 not including the 5${}^{\prime }$ terminal adenine A0. The two lateral loops (LL1 and LL2) are also indicated, as well as the diagonal loop (DL).

DNA and RNA sequences rich in Guanine (G) can fold in a Quadruple Helix (hereafter GQ), formed by stacks of four Gs arranged in a plane (tetrad) connected by Hoogsteen-like hydrogen bonds, and stabilized by the coordination of inner cations e.g., K${}^{+}$, Na${}^{+}$, etc. (see Figure 1) [1,2,3]. Despite these common features, GQs exhibit striking structural diversity. They can be formed both by the folding of a single nucleic acid strand, where the different tetrads are connected by loops, or by the coordination of several strands. Gs can be in syn or anti conformation with respect the glycosidic bond, and, consequently, there can be both parallel and antiparallel GQs. Considering that the loops can also adopt different structures, GQs can present many different folding topologies, depending on the sequence, the nature of the coordinated ions, and the temperature [1,3]. Moreover, supramolecular structures, involving more than one GQ, can be formed. Besides constituting a very stimulating challenge for structural biochemistry (both experimental and computational), the assessment of the static and dynamical conformational behavior of GQs is also extremely important from the biomedical point of view. Indeed, in the last years the detection of GQ occurrence in living cells has suggested that they might regulate key biological processes, such as telomere homeostasis, DNA replication, transcription, translation, and genomic instability [4]. The multiple biological functions of DNA and RNA GQs present opportunities to exploit GQ-related mechanisms for therapeutic intervention in various diseases [5,6,7,8]. In cancer, for instance, GQs are demonstrating their therapeutic potential as both anticancer drugs (particularly using aptamers) and targets (using GQ-targeted small molecule ligands able to interfere with telomeric functions and/or oncogene expression) [7,8]. The biological relevance of GQs motivates a deeper understanding of their conformational behaviour, which is however not a trivial task. In fact, crystallographic methods, besides selecting the conformation prone to crystallization, are not the best option to study flexible structures in solution. Optical spectroscopies, especially electronic circular dichroism (ECD) [9,10,11,12,13,14,15], have been shown to be particularly useful for the structural characterization of GQs, but they have a more limited atomic resolution. Integration with suitable computational methods in order to provide a greater atomic resolution is therefore very fruitful [13,16,17,18,19]. However, considering that a GQ contains, at least, a dozen closely stacked nucleobases, without considering the loops, a brute-force approach, exploiting full quantum mechanical (QM) calculations, is extremely computationally demanding. In this respect, the study of GQs folding topology has been predominantly conducted with the use of excitonic models, which have provided very useful elements for their assignment and interpretation [16,17,18,19].

A standard approach is to use a Frenkel excitonic Hamiltonian consisting of excitations localised on individual sites, and couplings between these local excitations (LEs). The couplings are typically calculated using the Coulombic interaction, in the simplest case adopting the point-dipole approximation, or more accurately adopting transition densities [20,21,22]. The Frenkel Hamiltonian with Coulombic coupling (FHC) approach has the advantage of being cost effective, due to only requiring the calculation of single site properties. However, for closely stacked chromophores (typically <4 Å) such as in GQs, wavefunction overlap effects and charge transfer (CT) states can become important, which the FHC approach does not include. This has been similarly highlighted by several recent studies on closely stacked Light Harvesting systems [23,24,25].

Within a theoretical framework similar to that proposed by Voityuk [26] and Tamura, Burghardt, and Polkehn [27,28,29,30,31,32,33], we have very recently introduced an approach to include these features, named the Fragment Diabatization Excitonic model (FrDEx) method [34]. It can include an arbitrary number of CT states, and the perturbation and overlap of LEs due to nearby chromophores via calculations on dimeric (or larger) units. Whilst this deviates from the single site calculations picture, one has the flexibility to choose to calculate only the important nearest neighbour CT states and wavefunction overlap effects. We tested FrDEx with two example GQ structures, illustrating its accuracy compared to a QM reference whilst being ∼3× quicker, and only ∼2× slower than an FHC approach.

The present study has two main goals. On the one hand, we want to further validate FrDEx for the study of a closely stacked Multi-Chromophore Assembly (MCA), using once more GQ as a critical stress test. To this aim, we shall compare the ECD spectra computed by FrDEx with those provided by full QM calculations, to better assess its performances with respect to standard excitonic models and define the most cost-effective approaches. At the same time, we shall use FrDEx to investigate some key issues for the study of GQ with spectroscopic methods, both computational and experimental. In particular, by using human telomeric sequence ((Tel21/GGG(TTAGGG)${}_{3}$, Tel22/AGGG(TTAGGG)${}_{3}$) as a test system, we shall investigate how the thermal fluctuations of quadruplex, studied here by Molecular Dynamics (MD) simulations, affect the spectra, as well as the role played by the adopted conformations of the loops in determining the spectral signal. Moreover, we shall study the sensitivity of the ECD spectra to the formation of supramolecular assemblies, formed by dimerization of two GQs. For this latter aim, we used FrDEx to compute the ECD spectrum of the monomeric and dimeric forms of a GQ forming sequence T30695 (5${}^{\prime }$TGGGTGGGTGGGTGGG3${}^{\prime }$), which has shown promising anti-cancer and anti-HIV activities [35,36]. It has been shown that T30695 can produce stable dimers, exhibiting the structure depicted in Figure 1c with two identical propeller-type parallel-stranded G-quadruplex subunits stacked via the 5${}^{\prime }$-5${}^{\prime }$ interface [36]. The comparison of FrDEx with the full QM and experimental spectra [37] will provide another stringent test for its reliability.

## 2. Methods

For FrDEx, an excitonic state *k* for a system of $N_{\mathrm{mol}}$ monomers is written as

(1) $$|\Psi^{k}\rangle =\sum_{m}^{N_{\mathrm{mol}}}\sum_{\alpha }^{N_{\mathrm{loc}}}C_{m}^{\alpha ,k}|L_{\alpha }^{m}\rangle +\sum_{m}^{N_{\mathrm{mol}}}\sum_{n\neq m}^{N_{\mathrm{mol}}}\sum_{\gamma }^{N_{\mathrm{CT}}}C_{mn}^{\gamma ,k}|{\mathrm{CT}}_{\gamma }^{m\to n}\rangle$$

where, for each monomer *m*, the index α labels the $N_{\mathrm{loc}}$ possible LEs ($L_{\alpha }^{m}$) with corresponding coefficient $C_{m}^{\alpha ,k}$. The index γ identifies the $N_{\mathrm{CT}}$ different types of CT states (${\mathrm{CT}}_{\gamma }^{m\to n}$) where an electron is transferred from monomer *m* to monomer *n*, with corresponding coefficient $C_{mn}^{\gamma ,k}$.

The FrDEx Hamiltonian on the basis of these LE and CT states is then written as:

(2) $$\begin{array}{cc} H_{\mathrm{FrDEx}}=\; & H_{\mathrm{intra}}+H_{\mathrm{inter}}\\ H_{\mathrm{intra}}= & \sum_{m}^{N_{\mathrm{mol}}}\left\{\sum_{\alpha }^{N_{\mathrm{loc}}}\left[\epsilon_{L_{\alpha }^{m}}|L_{\alpha }^{m}\rangle \langle L_{\alpha }^{m}|+\sum_{\beta \neq \alpha }^{N_{\mathrm{loc}}}V_{L_{\alpha }^{m}}^{L_{\beta }^{m}}\left(|L_{\alpha }^{m}\rangle \langle L_{\beta }^{m}|+h.c.\right)\right]\right\}\\ H_{\mathrm{inter}}= & \sum_{m}^{N_{\mathrm{mol}}}\sum_{n\neq m}^{N_{\mathrm{mol}}}\left\{\sum_{\alpha ,\beta }^{N_{\mathrm{loc}}}V_{L_{\alpha }^{m}}^{L_{\beta }^{n}}\left(|L_{\alpha }^{m}\rangle \langle L_{\beta }^{n}|+h.c.\right)\right.\\ & +\sum_{\gamma }^{N_{\mathrm{CT}}}\left[\epsilon_{{\mathrm{CT}}_{\gamma }^{m\to n}}|{\mathrm{CT}}_{\gamma }^{m\to n}\rangle \langle {\mathrm{CT}}_{\gamma }^{m\to n}|+\sum_{\delta \neq \gamma }^{N_{\mathrm{CT}}}V_{{\mathrm{CT}}_{\gamma }^{m\to n}}^{{\mathrm{CT}}_{\delta }^{m\to n}}\left(|{\mathrm{CT}}_{\gamma }^{m\to n}\rangle \langle {\mathrm{CT}}_{\delta }^{m\to n}|+h.c.\right)\right.\\ & +\sum_{\delta }^{N_{\mathrm{CT}}}V_{{\mathrm{CT}}_{\gamma }^{m\to n}}^{{\mathrm{CT}}_{\delta }^{n\to m}}\left(|{\mathrm{CT}}_{\gamma }^{m\to n}\rangle \langle {\mathrm{CT}}_{\delta }^{n\to m}|+h.c.\right)\\ & +\left.\left.\sum_{\alpha }^{N_{\mathrm{loc}}}\left(V_{L_{\alpha }^{m}}^{{\mathrm{CT}}_{\gamma }^{m\to n}}\left(|L_{\alpha }^{m}\rangle \langle {\mathrm{CT}}_{\gamma }^{m\to n}|+h.c.\right)+V_{L_{\alpha }^{m}}^{{\mathrm{CT}}_{\gamma }^{n\to m}}\left(|L_{\alpha }^{m}\rangle \langle {\mathrm{CT}}_{\gamma }^{n\to m}|+h.c.\right)\right)\right]\right\} \end{array}$$

where $H_{\mathrm{FrDEx}}$ is split into a intra-molecular part $H_{\mathrm{intra}}$ and an inter-molecular part $H_{\mathrm{inter}}$, and h.c. stands for Hermitian conjugate.

In the intra-molecular part, we have the LE energies $\epsilon_{L_{\alpha }^{m}}$, and the couplings of different LEs on the same monomer, $V_{L_{\alpha }^{m}}^{L_{\beta }^{m}}$. These couplings are zero in a monomer, but become non-zero in a MCA due to the electrostatic and polarisation effects of the surrounding monomers and/or the overlap with their molecular orbitals, causing LEs defined on an isolated monomer to mix when other monomers are present nearby. Furthermore, the $\epsilon_{L_{\alpha }^{m}}$ are typically equal to that found in the isolated monomer in ‘standard’ excitonic models, but they can also be influenced by the surrounding monomers in a MCA. In the following we will refer to both these phenomena as a ‘perturbation’ of the LEs.

In the inter-molecular part, we have the CT energies $\epsilon_{{\mathrm{CT}}_{\gamma }^{m\to n}}$; the couplings between LE and CT states $V_{L_{\alpha }^{m}}^{{\mathrm{CT}}_{\gamma }^{m\to n}}$ (the CT creates a hole in the same monomer involved in the LE), and $V_{L_{\alpha }^{m}}^{{\mathrm{CT}}_{\gamma }^{n\to m}}$ (the CT state transfers an electron to the same monomer involved in the LE); and the LE-LE couplings between LEs on different monomers, $V_{L_{\alpha }^{m}}^{L_{\beta }^{n}}$. The CT-CT couplings include those in which one CT state is transferring an electron from monomer *m* to monomer *n* and the other is transferring an electron from monomer *n* to monomer *m* with $V_{{\mathrm{CT}}_{\gamma }^{m\to n}}^{{\mathrm{CT}}_{\delta }^{n\to m}}$, and those between different types of CT states where they both transfer an electron from monomer *m* to *n* with $V_{{\mathrm{CT}}_{\gamma }^{m\to n}}^{{\mathrm{CT}}_{\delta }^{m\to n}}$.

These energies and couplings are calculated via a fragment diabatisation (FrD) technique, where we define diabatic states of some supramolecular complex (SC) consisting of $N_{\mathrm{frag}}$ fragments, a subset of the total $N_{\mathrm{mol}}$ monomers, using as reference states either the adiabatic states of the fragments (for LEs), or orbital transitions between the fragments (for CT states).

As derived and illustrated previously [34,38], the diabatic states |d〉 are then obtained by

(3) $$\begin{array}{cc} |d\rangle & =|a^{\mathrm{SC}}\rangle D\\ \; & =|a^{\mathrm{SC}}\rangle S^{T}{\left(SS^{T}\right)}^{-\frac{1}{2}} \end{array}$$

where $S=\langle R^{\mathrm{frags}}|a^{\mathrm{SC}}\rangle$ is the overlap of the reference states of the fragments ($|R^{\mathrm{frags}}\rangle$) with the adiabatic states of the SC ($|a^{\mathrm{SC}}\rangle$). The diabatic energies and couplings can then be calculated from the transformation matrix D applied to the diagonal matrix of adiabatic energies of the SC $H(a^{\mathrm{SC}})$

(4) $$H\left(d\right)=D^{T}H\left(a^{\mathrm{SC}}\right)D.$$

The flexibility of the FrDEx method lies in the ability to choose a SC of any size to compute the couplings and energies, so to balance computational cost and accuracy, avoiding, at the same time, any double-counting effect. Indeed, different sizes of SC may be chosen to parameterise $H_{\mathrm{intra}}$ and $H_{\mathrm{inter}}$, and as previous we choose pairs for $H_{\mathrm{inter}}$ [34], and test different sizes of $H_{\mathrm{intra}}$ in order to capture as much of the ‘perturbation’ effect of the LEs as possible.

In principle, any excited state electronic QM method can be used to parametrize FrDEx. However, the large size of the GQ systems studied here (containing at least 12 G bases) imposes the use of TD-DFT as reference QM method. The implementation of the FrD technique within the framework of TD-DFT has been presented previously, and we refer readers to these texts for further information [34,38].

For the calculation of the ECD spectra, we follow the procedure previously put forward by Jurinovich et al. [17,18,39,40], and used by us [34], to obtain an origin independent expression of the rotational strength in the velocity gauge $R^{v}$. See Supplementary Information, Section S1.1 for additional details.

### Computational Details

**Electronic calculations:** For our DFT/TD-DFT calculations, necessary to parametrize FrDEx and to provide the reference QM and FHC spectra, we selected the M05-2X functional [41], using a 6-31G(d) basis set, implemented within the Gaussian 16 package [42]. This functional can reliably describe CT transitions in stacked systems, as shown also by our previous studies on oligonucleotides [43]. Solvent effects of waTer have been included by the Polarizable Continuum Model (PCM) [44]. This approach has been already profitably used to the study the photoactivated behavior of oligonucleotides [43], including GQ [13,45,46,47,48]. The electronic couplings for the FHC approach were calculated using the ‘EET’ option within Gaussian, which utilises a transition density based approach to calculate the Coulombic couplings between LEs [22]. We also made selected test calculations with another functional, M06-2X [41], and a larger basis set to ensure that the ECD spectra are not qualitatively affected by these changes. These results are shown in the Supplementary Information, Figure S3.

**QM/MM:** For T30695, our reference QM results utilise a QM/MM model, applied to a structure obtained from PDB code 2LE6, without any further geometry optimization [36]. The QM region contains 12 G bases plus two inner Na${}^{+}$ ions for both the monomeric and the interface species and is described at the M05-2X/6-31G(d) level. The rest of the system is computed at the Molecular Mechanics (MM) level (amber force field parm96.dat) [49] and both regions are coupled using the ONIOM interface [50] as implemented in Gaussian [42]. The whole system is embedded in implicit water using PCM. An illustration of the QM/MM model is shown in the Supplementary Information, Figure S2.

**MD simulations:** Classical MD simulations in explicit solvent have been performed on Tel21 and Tel22 sequences by using the Amber16 package [51] and the OL15 [52] DNA force field. The GQ was solvated in a truncated octahedral box with a minimal distance of 10 Å of solute from the box border. We used SPC/E water model [53] and a 0.15 M excess NaCl. Two Na${}^{+}$ ions were manually inserted between the tetrads. Joung and Cheatham (JC) parameters for the ions were used [54]. The model 1 of the NMR structure with pdb code 143D [55] was chosen as starting structure for the simulations. The equilibration of the starting structure by using standard protocols was followed by a 1 microsec long single production run. Eleven structures extracted every 100 ns from the trajectory were selected for the ECD spectra calculations. The stacking interactions between G bases were assessed by calculating the distance between centres of mass of the bases as well as by evaluating the overlap areas of planar projections of the ring and exocyclic atoms in consecutive bases. The latter calculations have been performed by 3DNA software [56].

**FrDEx:** For all the FrDEx calculations, the sugar backbone and inner ions are removed from the GQ structures. For the majority of the computations, only the guanine bases are considered, where the guanine geometries from the PDB/MD are replaced by a 9-methyl-guanine, geometry optimised at the M05-2X/6-31G(d)/PCM(water) level of theory, by minimising the RMSD between them. We also made some tests by using directly the G structures provided by MD simulations. Analogously, in the study of the contribution of the loops, adenine and thymine are replaced by geometry optimised 9-methyladenine and 1-methylthymine, respectively.

The reference diabatic states for G include two $\pi \pi^{*}$ states (commonly referred to as L${}_{a}$ and L${}_{b}$) [57] for each base and either 4 or 12 CT states for each base pair, i.e., including one electron transitions between the frontier 1 or 2 occupied orbitals, and 2 or 3 virtual orbitals. For the parameterisation of $H_{\mathrm{inter}}$, we project the reference states onto 40 adiabatic states for all nearest-neighbour pairs. The nearest-neighbours are defined as the pairs that are immediately stacked on top of one another in adjacent tetrads, are hydrogen bonded in the same tetrad, or involved a guanine in one tetrad with a guanine that was hydrogen bonded to an immediately stacked guanine in an adjacent tetrad (e.g., G1-G5 or G1-G8 for Tel21 in Figure 1b).

For the parameterisation of $H_{\mathrm{intra}}$, we either use as the SC the already calculated pairs from the $H_{\mathrm{inter}}$ parameterisation and averaged over each base, or we performed an additional projection of only the L${}_{a}$ and L${}_{b}$ states onto 30 adiabatic states of a strand (e.g., G1-G2-G3 of Tel21 in Figure 1) or a tetrad (e.g., G2-G5-G8-G11 of Tel21 in Figure 1). An illustration is shown in the Supplementary Information, Figure S1. For the parameterisation of $H_{\mathrm{intra}}$ of the full T30695 GQ, the strands include 6 Gs, and we project onto 50 adiabatic states.

In order to allow an easier comparison with experiments and to consider all the sources of error in the reference QM calculation (functional, basis set, solvation model, absence of thermal and vibrational effects [58]) all of the computed spectra are red-shifted by −0.85 eV. This value is the shift enabling to superimpose the PCM/TD-M05-2X/6-31G(d) spectrum of 9-methylguanine in water onto the experimental one [34,57], and is similar to that necessary to obtain the same result for Adenine and Thymine bases. Since we always apply the same shift, we can check how our method reproduces the effect of the stacking geometry of the different folds/loops on the ECD spectra. All spectra are also phenomenologically broadened with a Gaussian with standard deviation of 0.21 eV, and calculated using a modified version of the EXAT code [18]. Further details are in the Supplementary Information, Section S1.1.

## 3. Results

### 3.1. Tel21

We first examine how some of the options available when using FrDEx, such as number of CT states and SC used to calculate the parameters for $H_{\mathrm{intra}}$, affect the computed spectra in Section 3.1.1. We then assess how the spectra depend on the geometry of individual Gs considered in the calculation (see Section 3.1.2) or the inclusion of the loop adenine and thymine bases (see Section 3.1.3). For these checks, we use either the NMR structure of Tel21, or representative snapshots from the Tel21 and Tel22 MD simulations. Finally, in Section 3.1.4 we combine an analysis of the snapshots extracted every 100 ns from the Tel21 MD simulations with FrDEx in order to gain insights on how thermal fluctuations modulate the ECD spectrum.

#### 3.1.1. Assessment of the FrDEx Options

**Figure 2:**
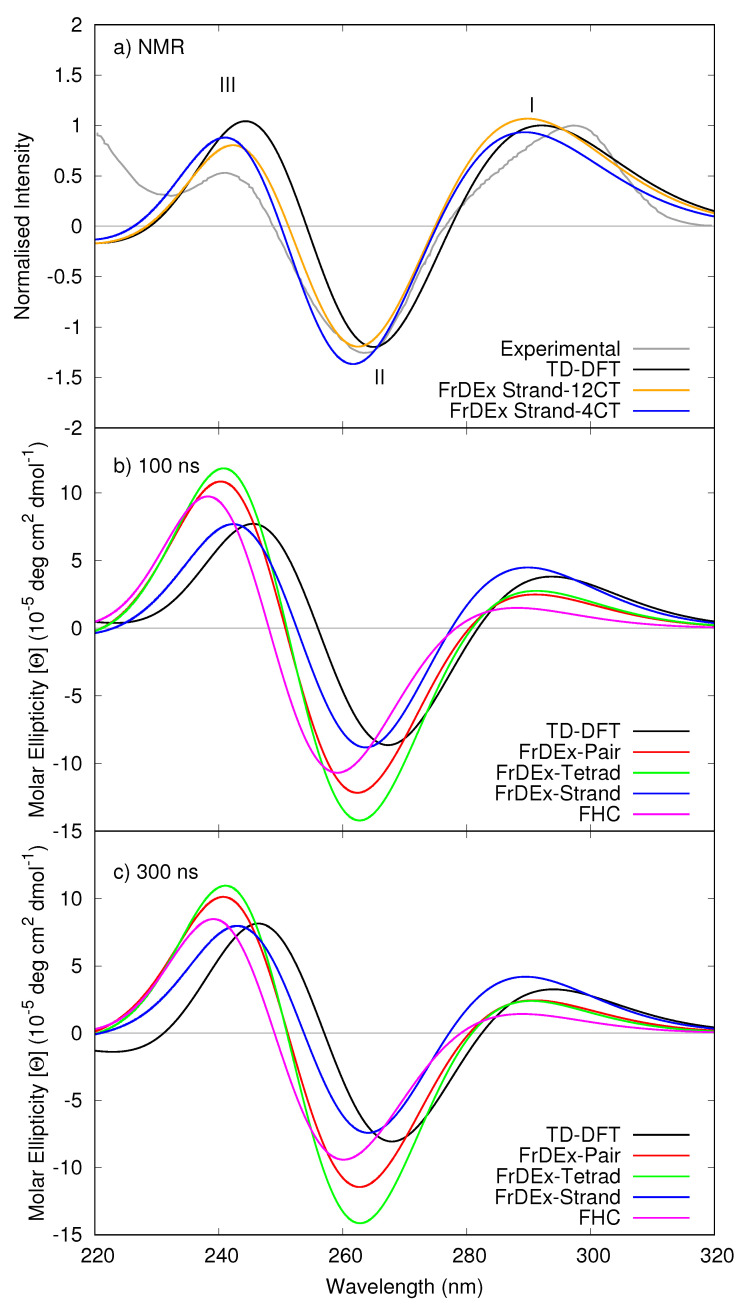
Computed ECD spectra of (**a**) Tel21 NMR structure by TD-DFT and FrDEx with 4 or 12 CT states, compared to experiment [59]. Panels (**b**,**c**) show computed spectra of Tel21 from MD snapshots at 100 and 300 ns, respectively, using TD-DFT and FrDEx with 4 CT states. All panels use pairs as the SC to calculate $H_{\mathrm{inter}}$, panel (**a**) uses a strand as SC to calculate $H_{\mathrm{intra}}$, whilst panels (**b**,**c**) use different sizes of SC indicated in the captions. In panel (**a**), the intensity of peak I is normalised to a value of 1 for both the experimental spectrum and the TD-DFT spectrum of the NMR structure, with the FrDEx spectra normalised using the same scaling factor as the TD-DFT spectrum. The spectra in panels (**b**,**c**) are not normalised. All calculated spectra are shifted by −0.85 eV.

In panel (a) of Figure 2 we report the ECD spectrum computed by TD-DFT and FrDEX (including either 4 or 12 CT states) for the NMR structure of Tel21, considering only G bases, with a strand as the SC for $H_{\mathrm{intra}}$, together with an experimental spectrum [59]. The intensity of the TD-DFT spectrum of Tel21 has been normalized to the lowest energy peak of the experimental CD, due to ambiguities in the measurement of nucleobase concentration in experimental spectra, as discussed in Ref. [60]. Moreover, we are interested in comparing the relative intensities of the peaks. The FrDEx spectra are normalised using the same value as the TD-DFT spectrum.

Once shifted, the TD-DFT spectrum of the NMR structure exhibits a positive lobe peaking at ∼290 nm (hereafter peak I), and a negative one, with similar intensity, with a maximum just above 260 nm (hereafter peak II). Finally there is another positive peak at ∼240 nm (peak III). In the following we mainly focus on peaks I and II, since the shape of peak III can be affected by higher energy transitions not included in our treatment. As previously discussed [34], the shape of the TD-DFT spectrum is in very good agreement with the experimental one, supporting the use of PCM/TD-M05-2X calculations to parametrize FrDEx to study GQ.

The FrDEx spectra compare well to the TD-DFT reference, and there is limited difference in choosing either 4 or 12 CT states, the latter of which was published in Ref. [34]. Therefore, for the remainder of the calculations in this work we include only 4 CT states, to ensure that the projection of diabatic states onto adiabatic states of the SC pair is as complete as possible when taking into account geometry fluctuations in the MD simulations that could destabilise the CT states.

Using two representative snapshots from the Tel21 MD simulations at 100 and 300 ns, we compare FrDEx calculations with different sizes of SC for for $H_{\mathrm{intra}}$ with TD-DFT and FHC results in panels (b) and (c) of Figure 2. Relative to the TD-DFT spectra of the snapshots, the FHC spectra are blue-shifted, and, more importantly, the relative intensity of the different peaks is not correctly predicted. In particular, peak I is significantly less intense than peak II (indeed, it is barely visible), whereas their intensity is more similar according to TD-DFT. On the contrary, independently of the approach used for *H*${}_{\mathrm{intra}}$, FrDEx spectra are much closer to the TD-DFT ones than the FHC ones. The blue-shift, though still present, is much smaller and the relative intensity of the three peaks are quite close to the TD-DFT ones. From the quantitative point of view, the strand spectra are, on average, those closest to the reference TD-DFT ones, as we also observed in our previous work [34].

#### 3.1.2. Guanine Internal Geometry

We have verified how our predictions change with the geometry assigned to each G base. In addition to the inter-molecular degrees of freedom, each base has its own vibrational motions, which affect the spectral shape associated with the individual electronic transitions. In our standard approach, this latter effect is missing, because we use for each G the geometry of the base optimized in water at the reference QM level (see Section 2.1).

**Figure 3:**
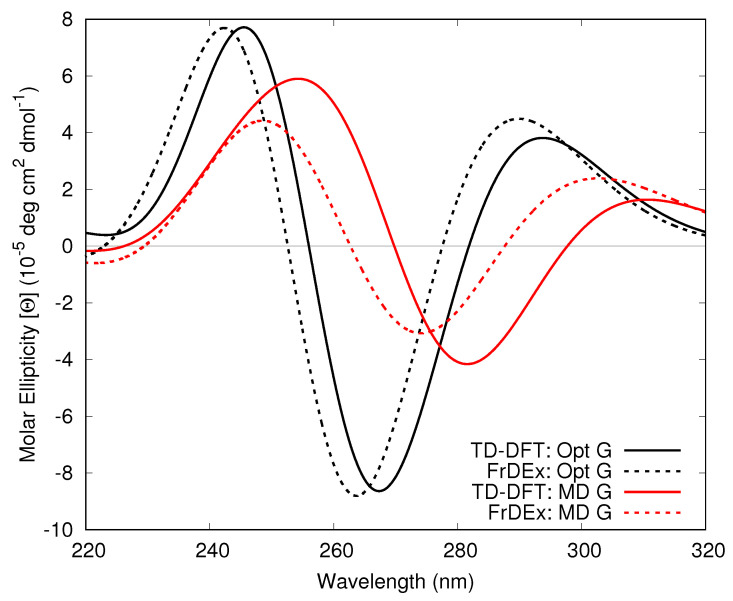
Computed ECD spectra of Tel21 GQ at 100 ns using as a geometry for G either the optimized structure of G monomer (black) or the geometry directly extracted from MD simulations (red). FrDEx spectra are reported as dashed lines and the full QM spectra as solid lines. All FrDEx calculations use pairs as the SC to calculate $H_{\mathrm{inter}}$ and strands for $H_{\mathrm{intra}}$. All spectra shifted by −0.85 eV.

Our test calculations on the Tel21 100 ns MD structure (see Figure 3) shows that the use of the MD geometries leads to a significant red-shift of the computed spectra. However, this is likely due to the well-known deficiencies of standard force fields (FFs) in accurately describing the intramolecular degrees of freedom. As discussed in previous papers [61], the overestimation of some C-C double bonds made by classical FF can lead to a smaller energy gap between occupied and virtual orbitals, and, therefore, to red-shifted electronic transitions. On the other hand, also in this case FrDEx spectra nicely match the full QM ones, showing that our method can also capture these effects, if more accurate FF are used in the MD simulations.

#### 3.1.3. Including Loop Bases

In our previous work and the results shown thus far, only the guanine bases have been included in the calculation of ECD spectra. However, the loops contain additional 9 bases (6 thymine and 3 adenine), which can be stacked among themselves and with the terminal guanines of each strand. These stacking effects could lead to modulation of the spectra, and it should be assessed whether this plays any significant role before moving onto thermal fluctuations of the guanine tetrads.

In Tel21 we can identify three loops, two we label as lateral (LL1 and LL2), and one Diagonal (DL) as shown in Figure 1b. Though all the loops obviously fluctuate during the dynamics, it is now assessed that the largest conformational variability is exhibited by the DL [62]. Here, we have considered the two most frequent conformations of the DL, which we label conf. 1 and conf. 2. The former exhibits an extensive stacking of bases T3/T4/A2/G7, whereas the latter shows stacking of T4/G1 and A2/G12, and, less frequently T3/A2 (Representative structures are shown in the Supplementary Information, Figure S5).

**Figure 4:**
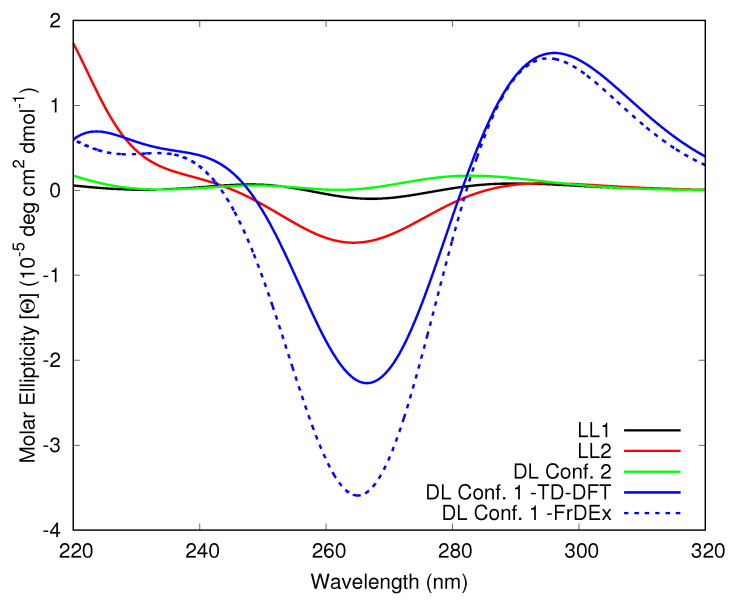
Computed ECD spectra of Lateral Loop 1 (LL1), Lateral Loop 2 (LL2) and Diagonal Loop (DL) in conf. 2 of the Tel21 100 ns structure and DL conf. 1 from Tel22 MD stucture at 100 ns. All spectra include only adenine and thymine bases, solid lines report TD-DFT spectra and dashed lines FrDEx spectra.

In the Tel21 100 ns structure the DL has conf. 2, whereas in the Tel22 100 ns structure it exhibits conf. 1. To evaluate whether these and the LL conformations resulted in any effect on the ECD spectra, first we calculated via TD-DFT the spectra including only the adenine or thymine bases, as shown in Figure 4. These calculations indicate that these bases in the LLs and DL in conf. 2 do not provide an intense signal. However, the stacking in conf. 1 does show a stronger bisignate peak at ∼300 nm (positive) and ∼265 nm (negative). Interestingly, this shape is nicely reproduced by FrDEx calculations.

**Figure 5:**
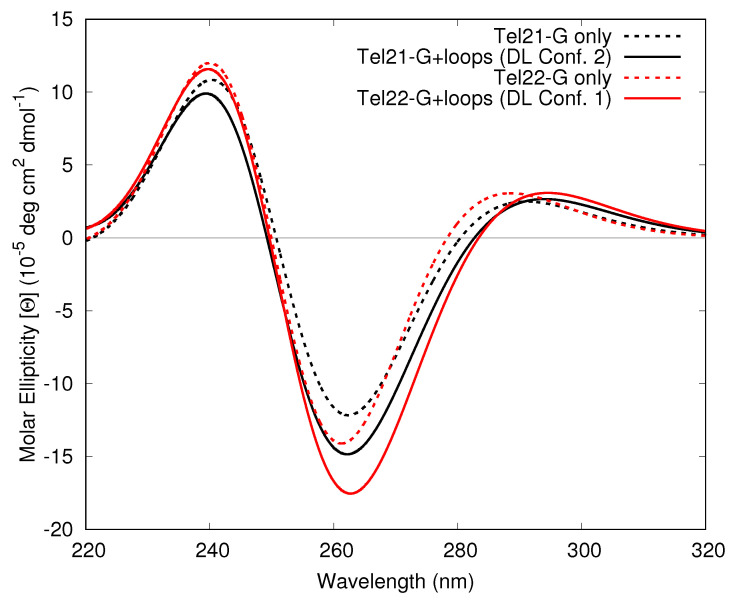
FrDEx computed ECD spectra including all adenine and thymine bases in the LLs and DL, and all G bases of Tel21 100 ns structure GQ with DL in conf. 2 (solid black) and Tel22 100 ns structure with DL in conf. 1 (solid red). Dashed lines indicate the spectra without the contributions from the loops, i.e., with only the G bases. We have not considered A0 base of Tel22 GQ. All FrDEx calculations use pairs as the SC to calculate $H_{\mathrm{inter}}$ and $H_{\mathrm{intra}}$. All calculated spectra are shifted by −0.85 eV.

Next, we have considered how the bases in the loops (adenine and thymine) affect the overall ECD spectra of Tel21, by using FrDEx to compute the ECD spectrum of the full system, including all Gs and the LLs and the DL in the two conformations previously discussed. This is shown in Figure 5, with the Tel21 100 ns snapshot with the DL in conf. 2 and the Tel22 100 ns snapshot with the DL in conf. 1. For the FrDEx calculations we use pairs as SC for both $H_{\mathrm{inter}}$ and $H_{\mathrm{intra}}$, due to possible unbalanced treatment of the central thymine base in the loops if we used a strand that incorporates the loop bases. We also do not consider the A0 base of Tel22. Inspection of Figure 5 shows that inclusion of the loop affects the spectra, although they remain quite similar to those obtained including only the G tetrads. The effects are more evident in the Tel22 100 ns structure exhibiting DL = conf. 1, where the maximum of the positive lobe I red-shifts by ∼10 nm, while the intensity of the negative lobe II increases.

#### 3.1.4. Considering Thermal Fluctuations

**Figure 6:**
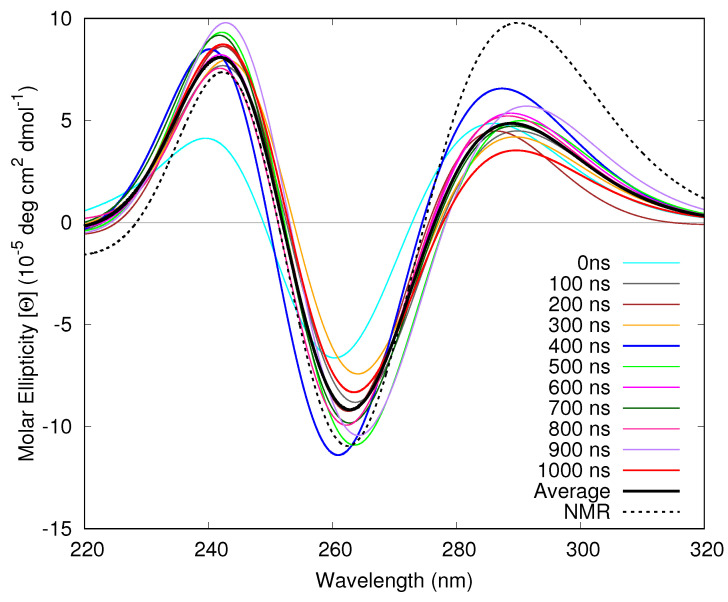
Computed ECD spectra of Tel21 GQ at the NMR structure and different time points on the MD Trajectory, as well as the average spectrum from the MD snapshots. All FrDEx calculations use strand as the SC to calculate $H_{\mathrm{intra}}$ and pair as the SC to calculate $H_{\mathrm{inter}}$. All calculated spectra are shifted by −0.85 eV.

Though NMR and ECD spectra show that the anti-parallel basket topology is largely predominant, at room temperature the Tel21 structure fluctuates around its equilibrium geometry. As the previous section illustrated that the adenine and thymine bases in the loops have only a modest effect on the ECD spectrum, and in order to disentangle these effects from the thermal fluctuations of the G bases, we will only consider the G bases when computing the spectra reported in the present section. In Figure 6, we show ECD spectra of Tel21 computed by FrDEx at structures obtained from snapshots at every 100 ns of the entire 1 μs MD trajectory.

The relative intensity of the different peaks significantly changes depending on the investigated structure. For example, peak I and II in the 1000 ns structure are, respectively, ∼3× and ∼1.5× less intense than in the 400 ns structure. Moreover, both peaks are red-shifted in the former structure. Comparing the spectra obtained from the MD snapshots to that obtained from the NMR structure, we observe that all the MD spectra have significantly lower intensities for peak I, and all but the 400 ns structure have slightly lower intensities for peak II. As a consequence, the spectrum obtained from averaging over all the spectra of the MD snapshots also has a much lower intensity peak I than the NMR structure spectrum, and a slightly lower intensity peak II. Furthermore, the peaks are slightly closer in energy in the averaged spectrum, mainly due to a blue shift of peak I.

**Figure 7:**
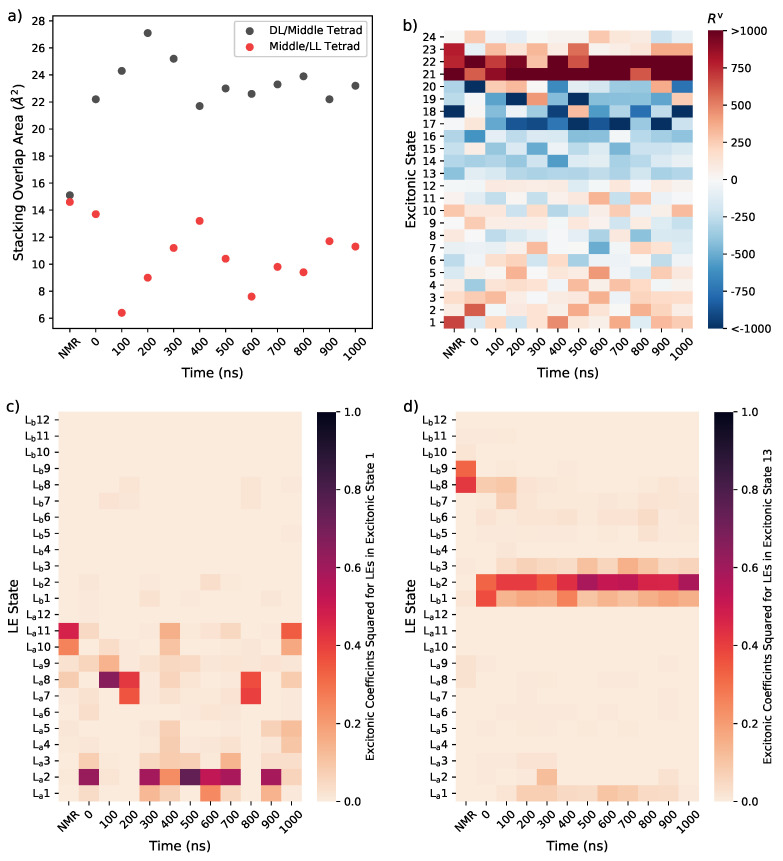
(**a**) Total overlap area between immediately stacked bases in adjacent tetrads, (**b**) rotational strengths of each excitonic state for each calculation of Figure 6 (**c**) coefficients of the L${}_{a}$/L${}_{b}$ states of the different G’s for excitonic state 1 (**d**) coefficients of the L${}_{a}$/L${}_{b}$ states of the different G’s for excitonic state 13.

Whilst a more complete conformational sampling would be required to determine whether there are differences in the NMR structure and that averaged from MD simulations, we can gain preliminary insights into the cause of these spectral changes through the analysis presented in Figure 7, verifying how the conformations adopted by the G bases affect the FrDEx spectra.

The analysis of the ten structures extracted from the MD trajectory highlights that changes can occur not only in the flexible loop regions, but also in the tetrad arrangement and stacking. From the structural data, we calculated the stacking overlap areas of pairs of adjacent G bases along a strand and then summed up the areas that contribute to the overlap between the ‘middle’ tetrad and that close to the DL (hereafter ‘DL’-tetrad), and the overlap between the middle tetrad and that close to the the LLs (hereafter ‘LL’-tetrad). Compared to the NMR structure, in the MD sampled structures the stacking area for ‘DL’/middle tetrad increases and the middle/‘LL’ one decreases, as shown in Figure 7a.

In Figure 7b we have plotted the rotational strength (responsible for the CD signal) associated with each of the 24 lowest excitonic states (i.e., the ones with predominant LE character) for each calculation in Figure 6. This shows that, on the average, excitonic state 1 and 13 (i.e., the ‘bottom’ of the L${}_{a}$ and L${}_{b}$ excitonic bands) provide a significant contribution to lowest energy positive and negative bands of the ECD signal, respectively. This is true especially for the NMR structure. In Figure 7c and d we have then reported the coefficients of the L${}_{a}$ and L${}_{b}$ LEs of each G in the excitonic states 1 and 13. In the NMR structure L${}_{a}$ states of G10 and G11 mainly contribute to excitonic state 1, whereas L${}_{b}$ states of G8 and G9 are the most important for Excitonic state 13. Both G10/11 and G8/G9 stacked pairs belong to the middle and ’LL’ tetrads, and are the pairs that contribute most to the stacking overlap of these tetrads (see Table S1 in the Supplementary Information).

On the contrary, with the partial exception of the 400 ns structure, in the structures extracted from the MD simulations, excitonic state 1 is dominated by L${}_{a}$ states of G1/G2 or G7/G8 pairs, whereas excitonic state 13 by the G1/G2 L${}_{b}$ states, i.e., from stacked pairs in the ‘DL’/Middle tetrads, which contribute a major part of their overlap (see Table S1 in the Supplementary Information). This result is consistent with our analysis of the MD simulations, which, when compared to the NMR structure, shows the stacking between the middle-’LL’ tetrad decreases and of middle-’DL’ tetrads increases. Furthermore, as shown in Figure S6 in the Supplementary Information, the distance between the centres of mass of the G8/G9 and G10/G11 pairs significantly increases from the NMR to MD structures, whilst simultaneously the distances between the centres of mass of the G1/G2 and G7/G8 pairs decreases.

**Figure 8:**
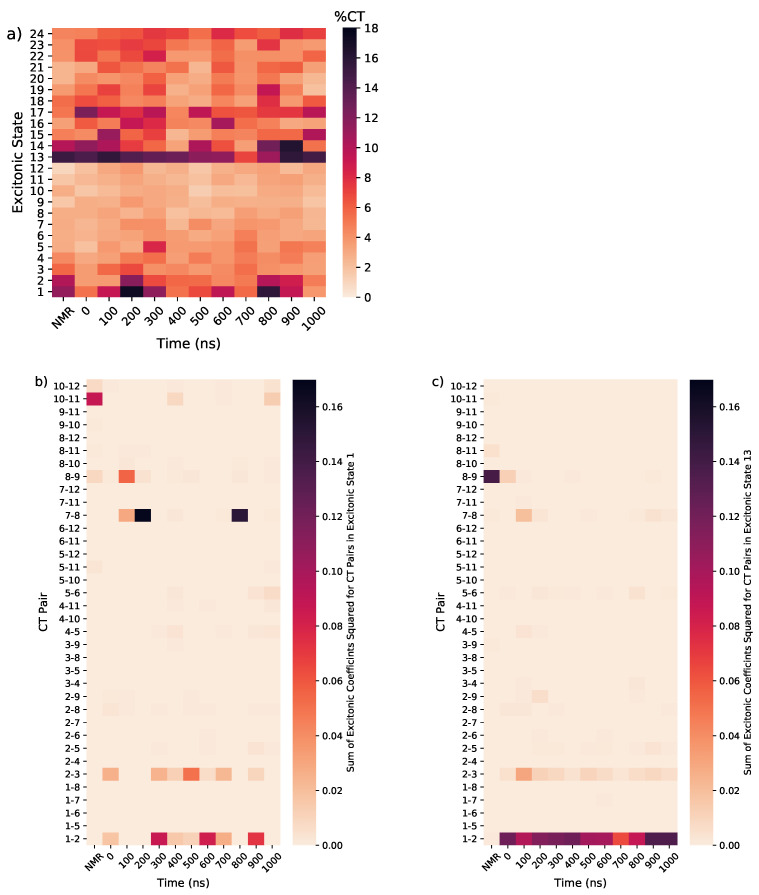
(**a**) Percentage involvement of CT states in the lowest 24 excitonic states for Tel21 in the NMR structure and MD snapshots; contribution of pairs of guanines to the CT states in excitonic state (**b**) 1 and (**c**) 13.

Interestingly, the excitonic states 1 and 13 also have significant CT character, in particular involving the same stacked pairs of guanines that provide the dominant L${}_{a}$/L${}_{b}$ character to these excitonic states, as shown in Figure 8. We hypothesise that an increase in stacking of a pair results in a stabilisation of the CT states and increased coupling with the LEs, resulting in a mixed LE/CT excitonic state at the bottom of the band.

### 3.2. Monomeric and Dimeric Forms of T30695

**Figure 9:**
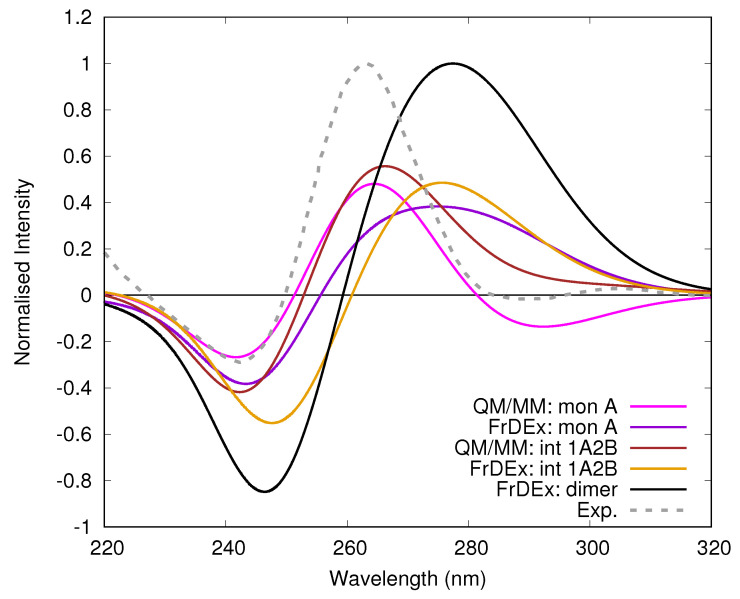
ECD spectra of the parallel GQ dimer T30695 from experiment [37] and computed by FrDEx, with low energy peaks normalised to 1. We also show the T30695 GQ “monomer” A, and the interface consisting of 1 tetrad of A and 2 of B (int 1A2B) computed by FrDEx and PCM/MM/TD-M05-2X/6-31G(d), both of which are normalised using the same value as the FrDEx spectrum of the dimer.

In Figure 9 we show normalised spectra of the T30695 GQ dimer (whose diagrammatic representation is shown in Figure 1c, from [37]), and computed by FrDEx. Also shown are spectra of the GQ formed from only monomer A of T30695 (i.e., containing only the G bases numbered with an ‘A’ in Figure 1), and that formed by the interface of 1 tetrad of monomer A and 2 tetrads of monomer B, computed by FrDEx and compared to MM/PCM/TD-M05-2X/6-31G(d) calculations. As we show in the Supplementary Information, Figure S4 the FrDEx spectra of monomer A and monomer B are equivalent, and the spectrum consisting of 2 tetrads of A and 1 of B is equivalent to that consisting of 1 tetrad of A and 2 of B.

The shape of the spectrum computed for the full dimer shows a strong positive peak at 262 nm and a negative one at 242 nm, in fair agreement with the experimental shape [37], considering that thermal fluctuations are not considered and that the intensity of the high-energy peak can be affected by higher-energy states not included in our treatment. From the quantitative point of view both computed peaks are more separated in energy than the experimental ones, and the intensity of the negative band is too large with respect to the positive one. We have registered similar trends also when studying another parallel GQ (i.e., the tetramolecular structure formed by TGGGGT sequence) [34]. It is possible that in the very closely stacked parallel GQ, the coupling between bases on different strands and/or from more distant tetrads (not considered in the calculations reported here in order to reduce the computational burden) is more important than in Tel21. Furthermore, additional CT couplings or higher lying states may also play a more important role than for Tel21. The fact that these parallel GQs only have two spectral bands compared to three for the anti-parallel GQs could make them more sensitive to these issues. On the other hand, considering the differences in the computational model used in the reference QM/MM calculations (see Section 2.1), the agreement between FrDEx and TD-DFT spectra appears satisfactory. For example, FrDEx captures the weak red-shift and the small increase in the intensity of the lowest energy positive peak of the ‘interface’ system, when compared to monomer A. These features give us more confidence in the FrDEx spectrum for the dimer system. Considering that in order to reproduce the spectrum up to 220 nm it would be necessary to compute a few hundred excited states, a full QM calculation of the spectrum would have an enormous computational cost.

## 4. Concluding Remarks

The study of the spectral properties and photoactivated dynamics of multichromophore assemblies is fundamental for many research fields of crucial scientific, industrial, and medical importance, including photosynthesis, solar energy, optoelectronics, and DNA photodamage. In this work, we have focussed on GQs, which have an important biological role and have also attracted significant attention for nanotechnology applications [63], due to their stability and self-assembly properties. In particular, we tackle the simulation of their ECD spectra. ECD spectroscopy is commonly used experimentally to quickly determine the folding topology exhibited by a GQ. The simulation of ECD spectra requires the use of QM calculations, and considering the large computational cost associated with the calculation of multichromophore systems, it has predominantly been undertaken with the use of excitonic Hamiltonians. In this context we chose GQ as a stringent test case for the application of FrDEx, a procedure to parametrize an excitonic Hamiltonian that we have very recently developed [34], which should be particularly suitable to treat closely stacked multichromophore systems.

The additional tests we have performed in the present study, concerning both the GQ core and the loops, fully confirm the potential of FrDEx to accurately simulate such systems. Indeed, for all the structures examined, FrDEx provides spectra fully consistent with that obtained by the reference QM calculations, and at the same time the method allows significant savings in the computational cost. Moreover, these tests confirm that FrDEx is more accurate than a standard excitonic approach, better suited to distant chromophores, as it includes LEs based on isolated monomers, and does not include charge transfer transitions. FrDEx, however, can include charge transfer transitions, which for small stacking distances can be significantly mixed with the LEs. Moreover, FrDEx can take into account the effect of the surrounding monomers on the LEs of each chromophore, so that they are not identical to the LEs of the isolated chromophore. Considering this effect in the parametrization of FrDEx (see for example the ’strand’ results) significantly increases the accuracy of the computed spectra. Finally, due to the separation of the Hamiltonian in intra- and inter-chromophore terms, the FrDEx procedure is particularly flexible and can be easily tuned to reach the desired compromise between accuracy and computational cost.

We used FrDEx to gain some insight into the effects that can modulate the spectral signal of GQ, such as the contribution of the loop bases (and therefore the effect of their conformational fluctuations), the effect of the thermal fluctuations in the stacking of the different tetrads, and the dimerization of the GQ. Though a complete assessment of these issues falls outside the scope of the present paper, since it would have required a much more accurate conformational sampling, our analysis has highlighted some interesting features in the spectroscopy. For example, we have shown how the conformation adopted of the loop can have a tiny, but non-vanishing, effect on the ECD spectral shape, and that fluctuations in the stacking distances of the different tetrads are mirrored by changes in the relative intensity of the different bands. Furthermore, related to this latter point, we illustrated how the changes in the stacking overlap area of the tetrads resulted in changes in the composition of the excitonic states at the bottom of the L${}_{a}$ and L${}_{b}$ bands. An increase in the overlap area between two tetrads led to greater involvement of both the L${}_{a}$/L${}_{b}$ and CT states of directly stacked guanines in these tetrads. It is possible that more general trends concerning the involvement of CT states on ECD spectra could be attributed, in the same manner as for absorption and photoluminescence spectra [64], and this is a promising avenue for future work.

Further developments of FrDEx, such as its implementation in a QM/MM scheme (particularly suitable to be coupled to MD simulations) or its extension to treat non-adiabatic dynamical processes in multichromophoric systems are planned, and investigations in this direction are underway.

## Data Availability

Additional computational details, ECD spectra, and MD analysis can be found in the Electronic Supporting Information.

## Supplementary Materials

The following are available online. Electronic circular dichroism spectra of DNA quadruple helices by using FrDEx excitonic model.

## Sample Availability

Samples of the compounds are not available from the authors.
