# Supplementary material for: Electronic Circular Dichroism Spectra of DNA Quadruple Helices Studied by Molecular Dynamics Simulations and Excitonic Calculations including Charge Transfer States
Source: Molecules. 2021 Aug 7;26(16):4789. doi: 10.3390/molecules26164789 (PMC8398971; doi:10.3390/molecules26164789)
Supplement: Supplementary file 1 [file molecules-26-04789-s001.zip › molecules-1298589-supplementary.pdf]

- The Name James Green should appear as "James A. Green"
- Similarly, the acronym next to the affiliations, and at the end of the manuscript with the author contributions should be "J. A. G." and the citation on the left hand side of p.1 "Green, J. A."
- Introduction, first sentence – we changed the sentence in "quadruple helix (GQ)"
- Introduction: add " (Tel21/GGG(TTAGGG)$_3$)" after "human telomeric sequence"
- Middle of page 2 "besides selecting **the** conformation"
- Just below, instead of "Integration with suitable computational methods is thus very fruitful" change with "Integration with suitable computational methods in order to provide a greater atomic resolution is therefore very fruitful"
- Page 3, second paragraph: instead of "we introduced" put "we have very recently introduced"
- Line below: "named the fragment diabatisation..." rather than just "the fragment diabatisation..."
- Page 3, third paragraph: instead of "by the loops" put "of the loops"
- Couple of lines below: instead of "To this aim," put "For this latter aim,"
- page 4 Fragment Diabatisation technique ---> Fragment Diabatisation (FrD) technique.
- Computational details - Electronic calculations "Solvent effects **of water**"
- page 5. MD simulation paragraph : Ten structure ---> eleven structures
- Page 6, second paragraph of FrDEx details: please change the marked sentence with: "or involved a guanine in one tetrad with a guanine that was hydrogen bonded to an immediately stacked guanine in an adjacent tetrad".
- Next paragraph "**the** already calculated pairs **from** the Hinter parameterisation and **averaged** over each base, or **we performed** an additional"
- Section 3.1: "For these checks we **use** either..." rather than "used".
- Page 7 figure 2 : Tel-21 ----> Tel21.
- Page 8, first sentence "exhibits" rather than "exhibited"
- Same paragraph, final word "to study GQs" rather than "to study GQ"
- Section 3.1.3 first sentence: Missing close of parenthesis, should read "LL2)"
- Same section, fourth paragraph: I think the sentence describing the full Tel21 system can be clarified a little more to "...full system **including all Gs and** the LLs and DL..."
- Figure 5, caption: missing space in "DL, and". Also in the caption, "Dashed lines **indicate**" is better.
- Section 3.1.4, first sentence: "at room temperature the Tel21 structure **fluctuates** around its equilibrium geometry"
- Section 3.1.4, second paragraph, first sentence: "...structure. For example," rather than "....structure, for example"
- Same paragraph, final sentence: "due to **a** blue shift"
- Section 3.2 first sentence: change ", shown in Figure 1c, from the experiment" to "(whose diagrammatic representation is shown in Figure 1c) from experiment"
- Figure 9 caption, second sentence: "We also show the T30695 GQ monomer A..."
- Section 3.2 second paragraph first sentence: "shows" rather than "showed"
- Section 3.2, final sentence split into two ending on "dimer system." and starting with "Considering that"
- Conclusions, second paragraph: rather than "including LEs based on isolated monomers" put "as it includes LEs based on isolated monomers"
